# Cochlear Function in Individuals with and without Spontaneous Otoacoustic Emissions

**DOI:** 10.3390/audiolres13050060

**Published:** 2023-09-05

**Authors:** Changgeng Mo, Bradley McPherson, Ting-Fung Ma

**Affiliations:** 1 Human Communication, Development, and Information Sciences, Faculty of Education, University of Hong Kong, Hong Kong SAR, China; dbmcpher@hku.hk; 2 Department of Statistics, University of South Carolina, Columbia, SC 29208, USA; tingfung@mailbox.sc.edu

**Keywords:** age, cochlear function, hearing, otoacoustic emissions

## Abstract

Purpose: This study investigated the status of spontaneous otoacoustic emissions (SOAEs) on cochlear function in a cohort of male/female participants with a wide age range. It examined whether there was a correlation between the presence of SOAEs and measurements of transient evoked otoacoustic emissions (TEOAEs), distortion product otoacoustic emissions (DPOAEs), SOAEs and extended high-frequency (EHF) hearing thresholds. Methods: 463 participants (222 male, 241 female; age range 20–59 years) with pure-tone thresholds ≤25 dB HL for octave frequencies of 500–8000 Hz were included in the study, divided into three age groups (20–29, 30–39, and 40–59 years). Evaluations included EHF (9000–16,000 Hz) hearing thresholds and TEOAE, DPOAE and SOAE measures. Results: Multiple regression models showed that participants with SOAEs had larger expected amplitudes and signal-to-noise ratios (SNRs) for TEOAE and DPOAE responses than participants without SOAEs, holding gender and age variables constant. Spearman correlation tests identified deterioration in TEOAE and DPOAE amplitudes and SNRs, and EHF hearing thresholds with age in participants without SOAEs. Among participants with SOAEs, no significant decreases in TEOAE and DPOAE measures were shown in participants with older age. Nonetheless, as expected, EHF hearing thresholds did become worse with age, with or without SOAEs. Conclusions: Participants with identifiable SOAEs had greater TEOAE and DPOAE amplitudes and SNRs than participants without SOAEs. SOAEs appear to be a useful marker of cochlear health in adults.

## 1. Introduction

Otoacoustic emissions (OAEs) are widely used in clinical audiology, as they provide rapid, non-invasive, objective assessments of outer hair cell function [1]. Spontaneous otoacoustic emissions (SOAEs) are sound signals from the inner ear that are not evoked by external stimuli. Otoacoustic emissions evoked by external stimuli are categorized as transient evoked otoacoustic emissions (TEOAEs) and distortion product otoacoustic emissions (DPOAEs). SOAEs, TEOAEs and DPOAEs are typically present in normal-hearing ears. However, the prevalence of SOAEs is lower than that of TEOAEs and DPOAEs, even in populations with normal hearing [2,3]. A number of studies have found that individuals with SOAEs have greater levels of TEOAE and DPOAE amplitude than those without observable SOAEs [2,4,5,6]. It has also been found that individuals with SOAEs have better extended high-frequency (EHF) hearing thresholds [4]. This may indicate that individuals with SOAEs have more active cochlear function.

Numerous factors influencing the prevalence of SOAE have been investigated. Well-known factors include age [7,8], gender [9], ear side [10], and hearing status [11]. The prevalence of SOAEs also has been found to vary by race [12,13]. Some previous studies had small numbers of participants, however, which may have led to insufficient statistical power for definitive conclusions to be made. Some studies have examined the effect of the presence of SOAEs on hearing status over a wide age range [2,14]. Other research has explored the effect of the presence of SOAEs on hearing status within a specific age range [4,6,15,16,17,18,19]. In the present study, SOAE prevalence was determined in 463 participants, as well as TEOAE amplitude and signal-to-noise ratio (SNR), DPOAE amplitude and SNR, and EHF hearing thresholds. The aim of the present study was to demonstrate the relationship between SOAE status and cochlear function with a large participant sample size. Additionally, this study aimed to examine the correlation between SOAEs and age, as well as cochlear function.

## 2. Methods

### 2.1. Participants

This study employed a quota sampling method based on convenience sampling, with the participants being university staff and students of Han Chinese ethnicity. Exactly 463 participants with clinically normal hearing, aged between 20 and 59 years (mean age was 28.5 years), were recruited. Participants were grouped according to their age into a 20–29 years group, a 30–39 years group and a 40–59 years group. All participants had pure-tone hearing thresholds of 25 dB HL or less at octave frequencies from 500 Hz to 8000 Hz, bilaterally. There were no reports of ear infection, chronic tinnitus, head injury or ear surgery history for any participants. All participant’s ears showed an otoscopic grade of 0 or +1 on the Sullivan scale [20], suggesting little or no cerumen in the ear canal. All had type A tympanograms [21], indicative of normal middle ear function. Prior to the study, all participants completed a written consent form. The study was conducted according to the guidelines of the Declaration of Helsinki and approved by the Human Research Ethics Committee at the University of Hong Kong (reference number: EA1909027).

### 2.2. Equipment and Procedure

Data collection was performed in a sound-treated room that met ANSI (American National Standard Institute) S3.1-1999 (R2013) standards. Pure-tone audiometry was conducted using a clinical audiometer (GSI AudioStar Pro, Eden Prairie, MN, USA) calibrated to ANSI S3.6-2010 with HDA300 headphones (Sennheiser, Wedemark, Germany). For OAE recordings, an ILO 292 II instrument with ILO v6 software (Otodynamics, London, UK) was utilized, and calibration was performed according to the manufacturer’s recommendations.

Participants first completed a case history form, which included personal information and noted self-reported hearing status. Then, participants received otoscopy and tympanometry, as well as conventional pure-tone audiometry and extended high-frequency (EHF) pure-tone audiometry. Lastly, TEOAEs, DPOAEs, and SOAEs were recorded. The measurement sequence of the left and right ears for each test was randomized. Data were obtained bilaterally.

To measure the hearing threshold of the participants, pure-tone audiometry was employed. Conventional Hughson–Westlake methods were used for the pure-tone audiometry procedure [22]. The EHF hearing thresholds were obtained at 9000 Hz, 10,000 Hz, 11,400 Hz, 12,500 Hz, 14,000 Hz and 16,000 Hz. All EHF hearing thresholds were also combined to obtain average EHF thresholds [4]. SOAEs were considered present when the absolute SOAE amplitude reached or exceeded −25 dB SPL and the signal amplitude exceeded all other spectral peaks in a 40 Hz range by 3 dB or more [11,23]. The current study used the default protocol provided by the ILO 292 device to obtain synchronized SOAE data. SOAEs were observed over a time window of 60–80 ms, and SOAE recordings stopped after 260 signal-averaging events [2,24]. A default, approximately 80 dB peSPL (peak equivalent SPL) click stimulus was used, along with a default averaging mode for TEOAE data recordings. TEOAE data were recorded in bands centered at 1000 Hz, 1400 Hz, 2000 Hz, 2800 Hz and 4000 Hz. A signal-to-noise ratio of >3 dB was accepted as a true TEOAE amplitude recording [12]. Reproducibility of >65% was considered indicative of a valid TEOAE response [12]. DPOAE measurement parameter settings of 2f1-f2 were used for DPOAE data recording in this study. The f2/f1 ratio was set to 1.22 with 65 dB SPL and 55 dB SPL for L1 and L2, respectively. A total of 14 f2 frequencies were recorded, ranging from 842 Hz to 7996 Hz. The recording stop criterion was defined as a minimum of three stimulus presentations per test frequency [25]. DPOAE recordings with an absolute amplitude greater than 0 dB SPL at each frequency were included in the analysis [26]. Absolute amplitude criteria were relevant to the physiological mechanism of DPOAE production, as it reflects the nonlinear distortion produced by the OHC in response to stimulus tones.

### 2.3. Data Analysis

All statistical data analyses were conducted using SPSS version 27.0 for Macintosh (IBM Corp., Armonk, NY, USA). Descriptive statistics included median TEOAE amplitude and SNR, DPOAE amplitude and SNR, and EHF hearing thresholds. To obtain average DPOAE results, the f2 frequencies were divided into low (841 HZ, 1001 Hz, 1184 Hz, 1416 Hz, 1685 Hz), middle (2002 Hz, 2380 Hz, 2832 Hz, 3369 Hz, 4004 Hz), high (4761 Hz, 5652 Hz, 6726 Hz, 7996 Hz) and overall (841 Hz–7996 Hz) average frequencies, and mean DPOAE amplitudes and SNR were determined over all data points in each frequency range. For TEOAE analysis, the total TEOAE amplitude and SNR are reported. The average extended high-frequency pure-tone threshold was calculated among frequencies from 9000 Hz to 16,000 Hz. TEOAEs, DPOAEs, and EHF hearing thresholds with different SOAE status (presence/absence) were compared after adjustment for age and gender, using multiple regression analysis. TEOAEs, DPOAEs, and EHF hearing thresholds with different SOAE status were the dependent variables, and age and gender were the independent variables. To determine possible correlations between age and each test (TEOAEs, DPOAEs, and EHF hearing thresholds), non-parametric Spearman rank tests were used. Participants were divided into three groups (20–29, 30–39, and 40–59 years) based on age. A Jonckheere–Terpstra test was used to compare the trend for the median at each condition [27]. The significance level was set at 0.05 for all analyses.

## 3. Results

### 3.1. Demographic Results

**Figure 1:**
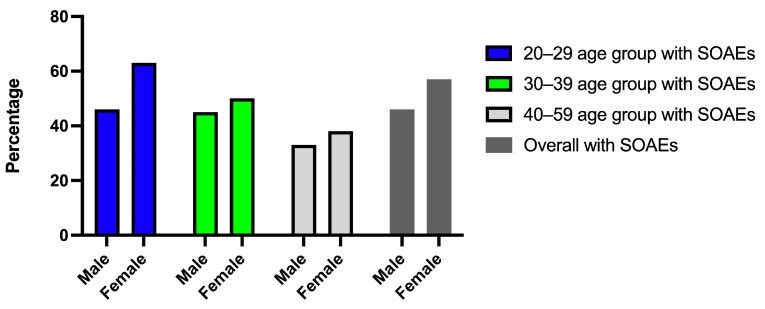
Percentage of SOAE presence for male and female participants within each age group.

There were 463 participants included in the analysis. There were approximately equal numbers of male and female participants in each age group (20–29 age group, male: 168, female: 161; 30–39 age group, male: 33, female: 48; 40–59 age group, male: 21, female: 32). Descriptive data on age, gender, participant number and SOAE prevalence are shown in Supplementary Table S1. Percentages of participants by Age, gender and SOAE prevalence are shown in Figure 1. Conventional audiometric information for participants with and without SOAEs in different age and gender groups is presented in the Supplementary Figures S1 and S2.

### 3.2. TEOAE Findings

**Figure 2:**
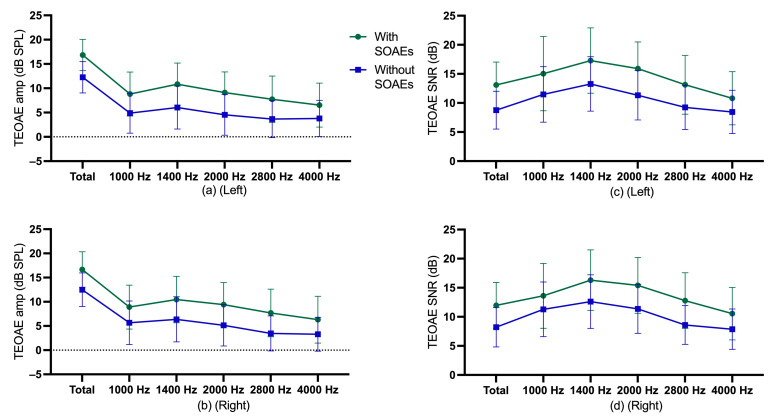
(**a**) mean and standard deviation of TEOAE amplitude of left ear for participants with and without SOAEs; (**b**) mean and standard deviation of TEOAE amplitude of right ear for participants without SOAEs; (**c**) mean and standard deviation of TEOAE SNR of left ear for participants with and without SOAEs; (**d**) mean and standard deviation of TEOAE SNR of right ear for participants without SOAEs.

Descriptive statistics for TEOAE amplitude and SNR of participants with and without SOAEs are shown in Figure 2, and descriptive statistics for gender and age group are shown in Supplementary Figure S3.

For the left ears, multiple regressions were performed for TEOAE amplitude and SNR in terms of age, gender, and SOAE status. The multiple regression model showed statistically significant in examining TEOAE amplitude, *F* = 77.71, *p* < 0.0005, adj R^2^ = 0.35. Age, gender, and SOAE status contributed statistically significantly to the model, *p* < 0.001. The multiple regression model was statistically significant in examining TEOAE SNR, *F* = 58.97, *p* < 0.0005, adj R^2^ = 0.29. Age, gender, and SOAE status contributed statistically significantly to the explanation, *p* < 0.001. Regression coefficients and standard errors can be found in the Supplementary Table S2.

For the right ears, multiple regressions were performed for TEOAE amplitude and SNR in terms of age, gender, and SOAE status. The multiple regression model was statistically significant in examining TEOAE amplitude, *F* = 59.49, *p* < 0.0005, adj R^2^ = 0.28. Age, gender, and SOAE status contributed statistically significantly to the explanation, *p* < 0.001. The multiple regression model was statistically significant in examining TEOAE SNR, *F* = 42.36, *p* < 0.0005, adj R^2^ = 0.22. Age, gender, and SOAE status contributed statistically significantly to the explanation, *p* < 0.001. Regression coefficients and standard errors can be found in the Supplementary Table S3.

**Figure 3:**
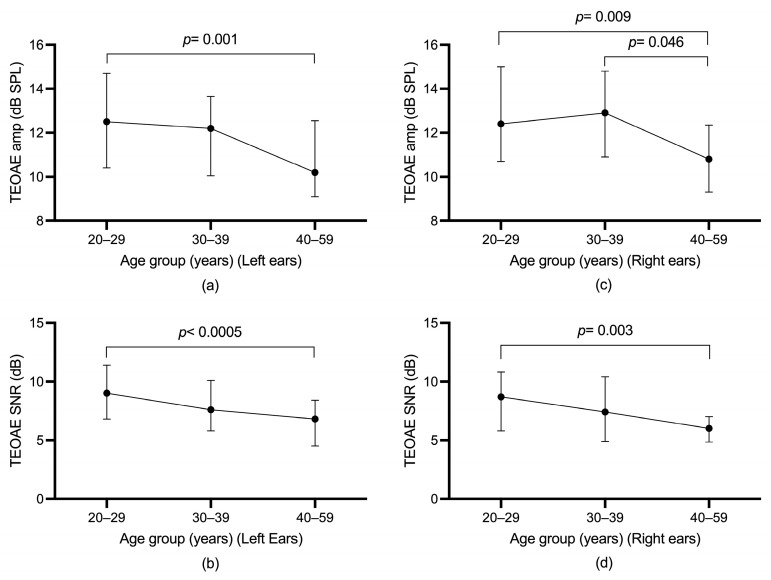
(**a**) median TEOAE amplitude of left ear across age group for participants without SOAEs; (**b**) median TEOAE SNR of left ear across age group for participants without SOAEs. (**c**) median TEOAE amplitude of right ear across age group for participants without SOAEs; (**d**) median TEOAE SNR of right ear across age group for participants without SOAEs. The upper and lower limits of the whiskers indicate the 75th and 25th percentiles.

**Figure 4:**
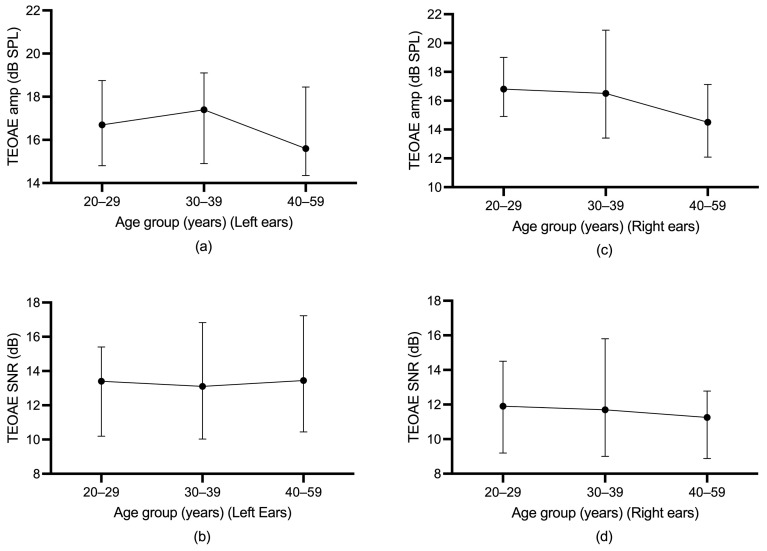
(**a**) median TEOAE amplitude of left ear across age group for participants with SOAEs; (**b**) median TEOAE SNR of left ear across age group for participants with SOAEs. (**c**) median TEOAE amplitude of right ear across age group for participants with SOAEs; (**d**) median TEOAE SNR of right ear across age group for participants with SOAEs. The upper and lower limits of the whiskers indicate the 75th and 25th percentiles.

Differences in TEOAE parameters (amplitude and SNR) among the three age groups were evaluated using the Jonckheere–Terpstra test. For participants without SOAEs, a statistically significant downward trend in total TEOAE amplitude in the left ear was found across age groups, *p* = 0.001, and the Spearman correlation between total TEOAE amplitude and age group was *r* = −0.195. A statistically significant downward trend in total TEOAE SNR in the left ear was also found across age groups, *p* < 0.0005, and the Spearman correlation between total TEOAE SNR and age group was *r* = −0.236. For participants without SOAEs, a statistically significant downward trend in total TEOAE amplitude in the right ear was found across age groups, *p* = 0.030), and the Spearman correlation between total TEOAE amplitude and age group was *r* = −0.141. A statistically significant downward trend in total TEOAE SNR in the right ear was also found across age groups, *p* = 0.004, and the Spearman correlation between total TEOAE SNR and age group was *r* = −0.186. For participants with SOAEs, however, a statistically significant trend in total TEOAE amplitude and total SNR over age group was not found. Figure 3 and Figure 4 illustrate the age trends for participants with or without SOAEs.

A Spearman correlation test was conducted to determine the age effect for TEOAEs in participants with SOAEs or without SOAEs. In general, the Spearman correlation test did not find significant results for participants with SOAEs in the left ears. For right ears, no significant results were found for participants with SOAEs. However, for participants without SOAEs, the Spearman correlation test found a significant negative correlation in TEOAE test parameters in the left and right ears. Supplementary Table S4 shows the results of the Spearman correlation test between TEOAEs and age for the left and right ears.

### 3.3. DPOAE Findings

**Figure 5:**
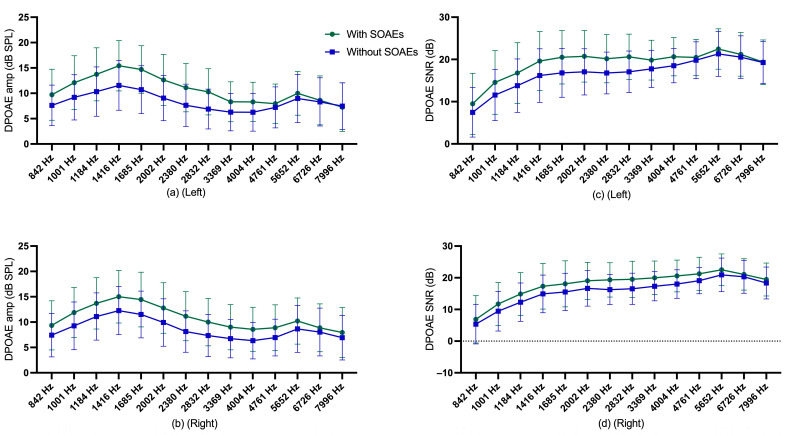
(**a**) mean and standard deviation of DPOAE amplitude of left ear for participants with and without SOAEs; (**b**) mean and standard deviation of DPOAE amplitude of right ear for participants without SOAEs; (**c**) mean and standard deviation of DPOAE SNR of left ear for participants with and without SOAEs; (**d**) mean and standard deviation of DPOAE SNR of right ear for participants without SOAEs.

Descriptive statistics for DPOAE amplitude and SNR of participants with and without SOAEs are shown in Figure 5, and descriptive statistics for gender and age group are shown in Supplementary Figures S4 and S5.

For the left ears, multiple regressions were performed for DPOAE amplitude and SNR at overall average frequency, low average frequency, middle average frequency, and high average frequency in terms of age, gender, and SOAE status. The multiple regression model was statistically significant in examining DPOAE amplitude (*F* = 26.18, *p* < 0.0005, adj R^2^ = 0.15) and SNR (*F* = 17.65, *p* < 0.0005, adj R^2^ = 0.10) at overall average frequency. Age and SOAE status contributed statistically significantly to the model, *p* < 0.0005. Similarly, for low average frequency, the multiple regression model was statistically significant in examining DPOAE amplitude (*F* = 22.94, *p* < 0.0005, adj R^2^ = 0.13) and SNR (*F* = 11.98, *p* < 0.0005, adj R^2^ = 0.08). Age and SOAE status contributed statistically significantly to the model, *p* < 0.01. For middle average frequency, the multiple regression model was statistically significant in examining DPOAE amplitude (*F* = 28.92, *p* < 0.0005, adj R^2^ = 0.17) and SNR (*F* = 23.06, *p* < 0.0005, adj R^2^ = 0.14). Age and SOAE status contributed statistically significantly to the model, *p* < 0.0005. For high average frequency, the multiple regression model was statistically significant in examining DPOAE amplitude (*F* = 4.52, *p* = 0.004, adj R^2^ = 0.04) and SNR (*F* = 3.67, *p* = 0.012, adj R^2^ = 0.03). Age contributed statistically significantly to the model, *p* < 0.0005. Regression coefficients and standard errors can be found in Supplementary Table S5.

For the right ears, multiple regressions were performed for DPOAE amplitude and SNR at overall average frequency, low average frequency, middle average frequency, and high average frequency in terms of age, gender, and SOAE status. The multiple regression model was statistically significant in examining DPOAE amplitude (*F* = 29.47, *p* < 0.0005, adj R^2^ = 0.16) and SNR (*F* = 17.34, *p* < 0.0005, adj R^2^ = 0.10) at overall average frequency. Age and SOAE status contributed statistically significantly to the model, *p* < 0.01. For low average frequency, the multiple regression model was statistically significant in examining DPOAE amplitude (*F* = 20.35, *p* < 0.0005, adj R^2^ = 0.11), age and SOAE status contributed statistically significantly to the model, *p* < 0.01. For DPOAE SNR at low average frequency, the multiple regression model was statistically significant in examining DPOAE SNR (*F* = 9.23, *p* < 0.0005, adj R^2^ = 0.05), SOAE status contributed statistically significantly to the model, *p* < 0.0005. For middle average frequency, the multiple regression model was statistically significant in examining DPOAE amplitude (*F* = 27.59, *p* < 0.0005, adj R^2^ = 0.16) and SNR (*F* = 19.95, *p* < 0.0005, adj R^2^ = 0.12). Age and SOAE status contributed statistically significantly to the model, *p* < 0.01. For high average frequency, the multiple regression model was statistically significant in examining DPOAE amplitude (*F* = 7.56, *p* < 0.0005, adj R^2^ = 0.05) and SNR (*F* = 5.91, *p* = 0.001, adj R^2^ = 0.04). Age and SOAE status contributed statistically significantly to the model, *p* < 0.05. Regression coefficients and standard errors can be found in Supplementary Table S6.

Spearman correlation tests were conducted to determine the age effect for DPOAEs in participants with SOAEs or without SOAEs. In general, the Spearman correlation tests did not find significant results for participants with SOAEs in left ears. For participants with SOAEs in right ears, significant negative correlations were found for DPOAE amplitude and SNR at middle average frequencies and high average frequencies. However, for participants without SOAEs, Spearman correlation tests found significant negative correlations for most DPOAE parameters (amplitude and SNR) in left and right ears. Table 1 and Table 2 show the results of Spearman correlation tests between DPOAEs and age for left and right ears.

Age differences among the three age groups were evaluated using the Jonckheere–Terpstra test. The results of average frequency, average low frequency, average mid frequency and average high frequency are presented in Table 3 and Table 4. For participants without SOAEs, a statistically significant downward trend in average frequency, average low frequency, average mid-frequency and average high-frequency DPOAE amplitudes and SNR was found across age groups. However, for participants with SOAEs, a significant downward trend was found at only a few frequencies, and no statistically significant trends in DPOAE amplitudes and SNR were found at most frequencies across age groups.

### 3.4. Extended High-Frequency Hearing Thresholds

**Figure 6:**
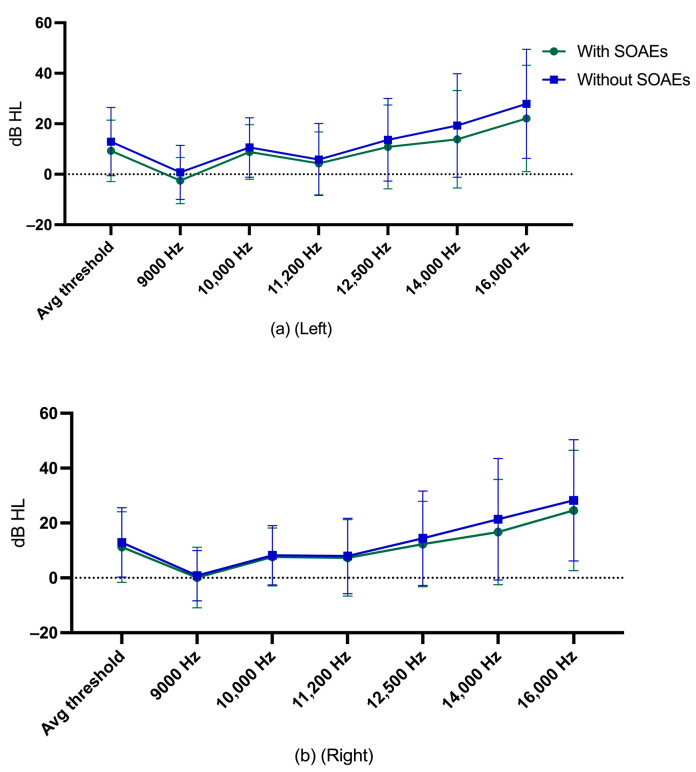
(**a**) mean and standard deviation of EHF hearing thresholds of left ear for participants with and without SOAEs; (**b**) mean and standard deviation of EHF hearing thresholds of right ear for participants without SOAEs.

Descriptive statistics for EHF hearing thresholds of participants with and without SOAEs are shown in Figure 6.

Multiple regression was run separately for the left and right ears to examine average EHF hearing thresholds from age, gender, and SOAE status. The multiple regression model was statistically significant in examining average EHF hearing threshold in left ears, *F* = 153.67, *p* < 0.0005, adj R^2^ = 0.50. Age contributed statistically significantly to the model, *p* < 0.0005. For the right ears, the multiple regression model was statistically significant in examining average EHF hearing threshold, *F* = 119.21, *p* < 0.0005, adj R^2^ = 0.44. Age contributed statistically significantly to the model, *p* < 0.0005. Regression coefficients and standard errors can be found in Supplementary Table S7.

Spearman’s correlation tests were used to examine the relationship between EHF hearing thresholds and age. All EHF hearing thresholds had a significant positive correlation with age. EHF hearing thresholds increased with age, regardless of the presence or absence of SOAEs. Table 5 and Table 6 show the results of Spearman’s correlation test for EHF hearing thresholds in left or right ears for participants with or without SOAEs.

Jonckheere–Terpstra tests were also used to assess trends across age groups in EHF hearing thresholds among participants with or without SOAEs. Findings were similar to Spearman’s correlation test results, with participants having higher EHF hearing thresholds with older age groups, regardless of whether they had SOAEs or not. Table 7 and Table 8 show the Jonckheere–Terpstra test results for participants with or without SOAEs in each EHF hearing threshold in left or right ears.

## 4. Discussion and Summary

The main finding of the current study was that participants with SOAEs had greater cochlear function (in terms of OAE amplitudes) compared to those without SOAEs. Furthermore, a statistically significant negative correlation between TEOAEs/DPOAEs and age was found in this study for participants without SOAEs. Jonckheere–Terpstra test results supported this finding, as participants without SOAEs in the 20–29 age group had better TEOAEs/DPOAEs than participants without SOAEs in the older age group. However, similar results were not found in participants with SOAEs. No statistically significant age-related correlations were found for TEOAEs among participants with SOAEs. Jonckheere–Terpstra test results also confirmed this finding, with no significant differences found between age groups among participants with SOAEs. For DPOAEs, however, a significant negative correlation was found between right-ear DPOAEs and age in participants with SOAEs, while no such negative correlation was found for the left-ear DPOAEs. One possibility for this asymmetry in DPOAEs is that the right ear has a closer potential source of noise related to blood flow than the left ear [28,29]. For EHF hearing thresholds, significantly elevated hearing thresholds were observed mainly in left ear for participants without SOAEs. Significant negative age-related EHF hearing thresholds were found for all participants, including the average EHF hearing threshold. Jonckheere–Terpstra test results confirmed similar findings, with the older age group having higher EHF hearing thresholds than the younger age group in all participants (with or without SOAEs). There are competing theories as to why DPOAE and TEOAE results differed in relation to age with different SOAE status. According to reflection theory, TEOAEs and SOAEs may have similar production mechanisms, and the presence of SOAEs may contribute to the preservation of TEOAEs with age; according to oscillator theory, DPOAEs and SOAEs may have different production mechanisms, and the presence of SOAEs may not affect DPOAEs with age [30].

Similar to previous studies, this study found that participants with SOAEs had greater TEOAE amplitude and SNR than those without SOAEs, even after controlling for age and gender variables [2,15,31]. This study confirmed that there were greater TEOAE amplitude and SNR in participants with SOAEs in a larger participant sample. In addition, this study also found no statistically significant negative correlation between TEOAEs and age in participants with SOAEs. In contrast, a significant negative correlation between TEOAEs and age was found for participants without SOAEs. Similar results were found for the Jonckheere–Terpstra test, with a significant downward trend in TEOAEs across age groups for participants without SOAEs and no downward trend found for participants with SOAEs.

Results from a multiple regression model revealed that participants with SOAEs had larger expected values in DPOAEs than those without SOAEs, holding the gender and age variables constant. In line with a large number of previous studies, this study observed that participants with SOAEs had better DPOAEs than participants without SOAEs [4,5,6,18,19]. However, the present study observed a significant negative correlation with age for most of the frequency range of DPOAEs in participants without SOAEs and a significant negative correlation in only some of the frequency range on right ears in participants with SOAEs. Jonckheere–Terpstra test results confirmed a downward trend in DPOAEs for participants without SOAEs across age group comparisons. For participants with SOAEs, a significant downward trend was found only in average frequency, average mid-frequency and average high-frequency DPOAEs in right ears.

For the extended high-frequency hearing thresholds, there was no statistically significant relationship between participants with SOAEs and without SOAEs, regardless of ear laterality, holding the age and gender variables constant. This study also confirms that the age variable has a greater impact on EHF hearing thresholds, regardless of SOAEs and gender. Both the Spearman correlation test and the Jonckheere–Terpstra test showed significant negative correlations with age and significant age-related downward trends in participants with and without SOAEs. This finding is consistent with the findings of Avan et al. [32] but contrary to the findings of Schmuziger et al. [4]. Avan et al. [32] included 43 participants between the ages of 24 and 50, while Schmuziger et al. [4] included 57 participants between the ages of 16 and 19. The present study has a wider age range and larger sample size, and it can be inferred in conjunction with previous studies that the age factor has a more significant effect on EHF hearing thresholds than on SOAE prevalence.

The findings of the current study suggest that participants with the presence of SOAEs may be less affected by aging in relation to cochlear function than those without SOAEs. A large body of evidence demonstrates that the prevalence of SOAE varies with age, with older age implying a lower prevalence of SOAE [2,7,14,16]. This study found no significant age-related correlation for participants with SOAEs on TEOAEs and DPOAEs. Jonckheere–Terpstra tests revealed no significant age-related trends for TEOAEs and DPOAEs among participants with SOAEs. This may imply that SOAEs can be used as a biomarker of cochlear health in the adult population. A limitation of this study was that the number of older age group participants was small, and more elderly participants with SOAEs should be entered into future studies. For SOAEs, the age factor plays an important role, and the inclusion of infant and adolescent age groups also should be considered in future studies. In addition, it was possible that the 5 dB increment step in routine clinical audiometry was not sufficiently sensitive for optimal hearing threshold comparisons between the SOAE+ and SOAE− groups. Follow-up studies measuring audiogram fine structure (Békésy or 1 dB step audiometry) should be considered and may elicit differences between SOAE+ and SOAE− groups. Effective collection of SOAE data appears to provide accurate and objective supplementary information on cochlear function over a wide range of frequencies [4].

## Figures and Tables

**Table 1:** Spearman correlation test results between DPOAE amplitude/SNR and age in left ears.

	With SOAEs		Without SOAEs	
Test Parameter	Spearman Correlation	Significance	Spearman Correlation	Significance
Avg low frequency amplitude	−0.081	NS	−0.187	**
Avg low frequency SNR	0.010	NS	−0.184	**
Avg mid frequency amplitude	−0.111	NS	−0.226	***
Avg mid frequency SNR	−0.113	NS	−0.203	**
Avg high-frequency amplitude	−0.123	NS	−0.174	**
Avg high-frequency SNR	−0.111	NS	−0.170	**
Avg frequency amplitude	−0.146	NS	−0.238	***
Avg frequency SNR	−0.064	NS	−0.232	***

Note: NS indicates not significant, asterisk ** indicates *p* < 0.001, asterisk *** indicates *p* < 0.0005; SNR indicates signal-to-noise ratio.

**Table 2:** Spearman correlation test results between DPOAE amplitude/SNR and age in right ears.

	With SOAEs		Without SOAEs	
Test Parameter	Spearman Correlation	Significance	Spearman Correlation	Significance
Avg low frequency amplitude	−0.022	NS	−0.213	**
Avg low frequency SNR	0.022	NS	−0.199	**
Avg mid frequency amplitude	−0.213	**	−0.170	*
Avg mid frequency SNR	−0.154	*	−0.161	*
Avg high-frequency amplitude	−0.190	**	−0.156	*
Avg high-frequency SNR	−0.160	*	−0.128	NS
Avg frequency amplitude	−0.151	*	−0.267	***
Avg frequency SNR	−0.085	NS	−0.240	***

Note: NS indicates not significant, asterisk * indicates *p* < 0.05, asterisk ** indicates *p* < 0.001, asterisk *** indicates *p* < 0.0005; SNR indicates signal-to-noise ratio.

**Table 3:** Jonckheere–Terpstra test results for DPOAE amplitudes and SNRs in left ear.

	With SOAEs		Without SOAEs	
Test Parameter	Correlation	Significance	Correlation	Significance
Avg frequency amplitude	−0.064	NS	−0.231	***
Avg frequency SNR	−0.018	NS	−0.225	***
Avg low frequency amplitude	−0.039	NS	−0.181	**
Avg low frequency SNR	0.008	NS	−0.202	***
Avg mid frequency amplitude	−0.0065	NS	−0.211	***
Avg mid frequency SNR	−0.076	NS	−0.176	*
Avg high-frequency amplitude	−0.159	NS	−0.181	*
Avg high-frequency SNR	−0.133	NS	−0.160	*

Note: NS indicates not significant, asterisk * indicates *p* < 0.05, asterisk ** indicates *p* < 0.001, asterisk *** indicates *p* < 0.0005; SNR indicates signal-to-noise ratio. NS indicates not significant; SNR indicates signal-to-noise ratio.

**Table 4:** Jonckheere–Terpstra test results for DPOAE amplitudes and SNRs in right ears.

	With SOAEs		Without SOAEs	
Test Parameter	Spearman Correlation	Significance	Spearman Correlation	Significance
Avg frequency amplitude	−0.143	*	−0.231	***
Avg frequency SNR	−0.086	NS	−0.250	***
Avg low frequency amplitude	−0.014	NS	−0.200	*
Avg low frequency SNR	0.029	NS	−0.195	*
Avg mid frequency amplitude	−0.201	*	−0.193	*
Avg mid frequency SNR	−0.140	*	−0.181	*
Avg high-frequency amplitude	−0.165	*	−0.153	*
Avg high-frequency SNR	−0.147	*	−0.132	NS

Note: NS indicates not significant, asterisk * indicates *p* < 0.05, asterisk *** indicates *p* < 0.0005; SNR indicates signal-to-noise ratio. NS indicates not significant; SNR indicates signal-to-noise ratio.

**Table 5:** EHF hearing thresholds in left ears.

			Avg. EHF	9000 Hz	10,000 Hz	11,200 Hz	12,500 Hz	14,000 Hz	16,000 Hz
With SOAE	Age	Spearman correlation	0.487	0.216	0.301	0.356	0.386	0.481	0.529
		Significance	***	**	***	***	***	***	***
Without SOAE	Age	Spearman correlation	0.504	0.273	0.371	0.351	0.417	0.484	0.550
		Significance	***	***	***	***	***	***	***

Note: asterisk ** indicates *p* < 0.001, asterisk *** indicates *p* < 0.0005; SOAE indicates spontaneous otoacoustic emission.

**Table 6:** EHF hearing thresholds in right ears.

			Avg. EHF	9000 Hz	10,000 Hz	11,200 Hz	12,500 Hz	14,000 Hz	16,000 Hz
With SOAE	Age	Spearman correlation	0.440	0.260	0.310	0.401	0.396	0.413	0.454
		Significance	***	***	***	***	***	***	***
Without SOAE	Age	Spearman correlation	0.508	0.329	0.368	0.397	0.281	0.464	0.531
		Significance	***	***	***	***	***	***	***

Note: asterisk *** indicates *p* < 0.0005; SOAE indicates spontaneous otoacoustic emission.

**Table 7:** Jonckheere–Terpstra test results for EHF hearing thresholds in left ears.

		Avg. EHF	9000 Hz	10,000 Hz	11,200 Hz	12,500 Hz	14,000 Hz	16,000 Hz
With SOAE	Significance	***	***	***	***	***	***	***
	Correlation	0.502	0.369	0.378	0.364	0.404	0.464	0.500
Without SOAE	Significance	***	***	***	***	***	***	***
	Correlation	0.469	0.321	0.412	0.417	0.386	0.437	0.505

Note: asterisk *** indicates *p* < 0.0005; SOAE indicates spontaneous otoacoustic emission.

**Table 8:** Jonckheere–Terpstra test results for EHF hearing thresholds in right ears.

		Avg. EHF	9000 Hz	10,000 Hz	11,200 Hz	12,500 Hz	14,000 Hz	16,000 Hz
With SOAE	Significance	***	***	***	***	***	***	***
	Correlation	0.483	0.366	0.375	0.439	0.332	0.480	0.495
Without SOAE	Significance	***	***	***	***	***	***	***
	Correlation	0.425	0.336	0.291	0.366	0.363	0.417	0.440

Note: asterisk *** indicates *p* < 0.0005; SOAE indicates spontaneous otoacoustic emission.

## Data Availability

The data sets analyzed in the current study can be obtained from the corresponding author by interested researchers upon reasonable request.

## Supplementary Materials

The following supporting information can be downloaded at: https://www.mdpi.com/article/10.3390/audiolres13050060/s1, Table S1: Prevalence of SOAEs by age and gender; Table S2: Regression coefficients and standard errors for TEOAE amplitude and SNR in left ears in terms of age, gender, and SOAE status; Table S3: Regression coefficients and standard errors for TEOAE amplitude and SNR in right ears in terms of age, gender, and SOAE status; Table S4: Spearman correlation test results between TEOAEs and age in left and right ears; Table S5: Regression coefficients and standard errors for DPOAE amplitude and SNR (at overall average frequencies, average low frequencies, average mid frequencies and average high frequencies) in left ears in terms of age, gender, and SOAE status; Table S6: Regression coefficients and standard errors for DPOAE amplitude and SNR (at overall average frequencies, average low frequencies, average mid frequencies and average high frequencies) in right ears in terms of age, gender, and SOAE status; Table S7: Regression coefficients and standard errors for extended high frequency hearing threshold in left and right ears in terms of age, gender, and SOAE status; Figures S1 and S2: Conventional audiometric information for participants with and without SOAEs in different age and gender groups; Figure S3: Descriptive statistics for TEOAE amplitude and SNR of participants with and without SOAEs in gender and age groups; Figures S4 and S5: Descriptive statistics for DPOAE amplitude and SNR of participants with and without SOAEs in age and gender groups.
