# Supplementary material for: Cochlear Function in Individuals with and without Spontaneous Otoacoustic Emissions
Source: Audiol Res. 2023 Sep 5;13(5):686–99. doi: 10.3390/audiolres13050060 (PMC10514821; doi:10.3390/audiolres13050060)
Supplement: Supplementary file 1 [file audiolres-13-00060-s001.zip › audiolres-2485077-supplementary.pdf]

Supplementary material

Supplementary table S1:

Table S1. Prevalence of SOAEs by age and gender.

	**Participants with SOAE**	**Participants without SOAE**
Overall sample	236 (51%)	227 (49%)
Male	99 (45%)	123 (55%)
Female	137 (57%)	104 (43%)
20-29 year age group	178 (54%)	151 (45%)
Male	77 (46%)	91 (54%)
Female	101 (63%)	60 (37%)
30-39 year age group	39 (48%)	42 (52%)
Male	15 (45%)	18 (55%)
Female	24 (50%)	24 (50%)
40-59 year age group	19 (36%)	34 (64%)
Male	7 (33%)	14 (67%)
Female	12 (38%)	20 (62%)

Supplementary Figure S1:

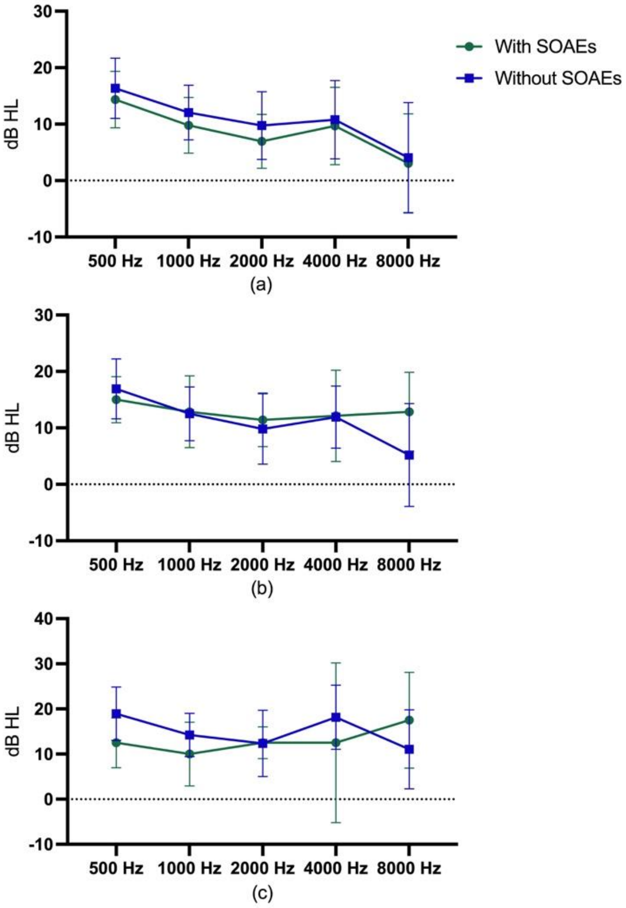

Figure S1. a, mean and standard deviation of pure tone threshold of left ear of male participants with and without SOAEs in 20-29 age group; b, mean and standard deviation of pure tone threshold of left ear of male participants with and without SOAEs in 30-39 age group; c, mean and standard deviation of pure tone threshold of left ear of male participants with and without SOAEs in 40-59 age group.

Supplementary Figure S2:

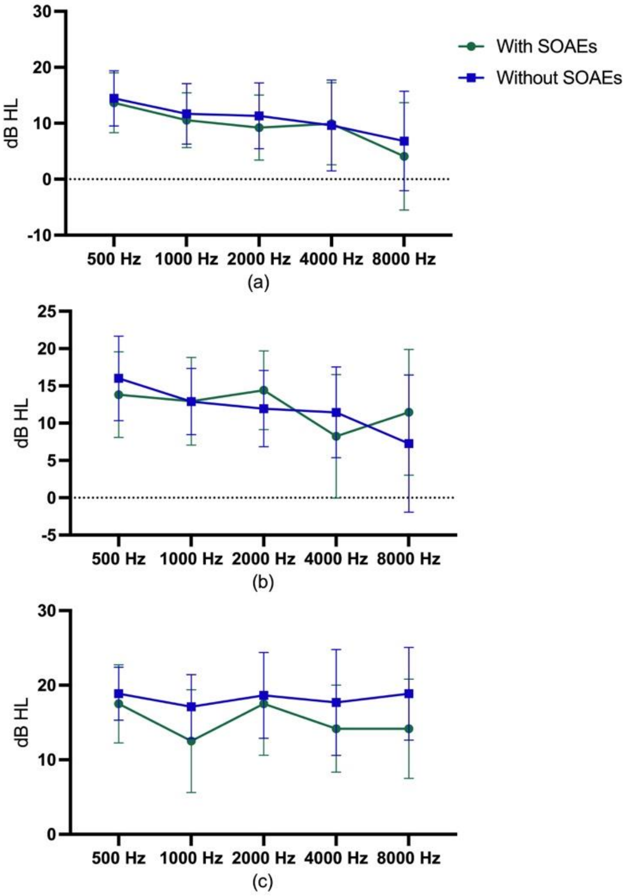

Figure S2. a, mean and standard deviation of pure tone threshold of left ear of female participants with and without SOAEs in 20-29 age group; b, mean and standard deviation of pure tone threshold of left ear of female participants with and without SOAEs in 30-39 age group; c, mean and standard deviation of pure tone threshold of left ear of female participants with and without SOAEs in 40-59 age group.

Supplementary Figure S3:

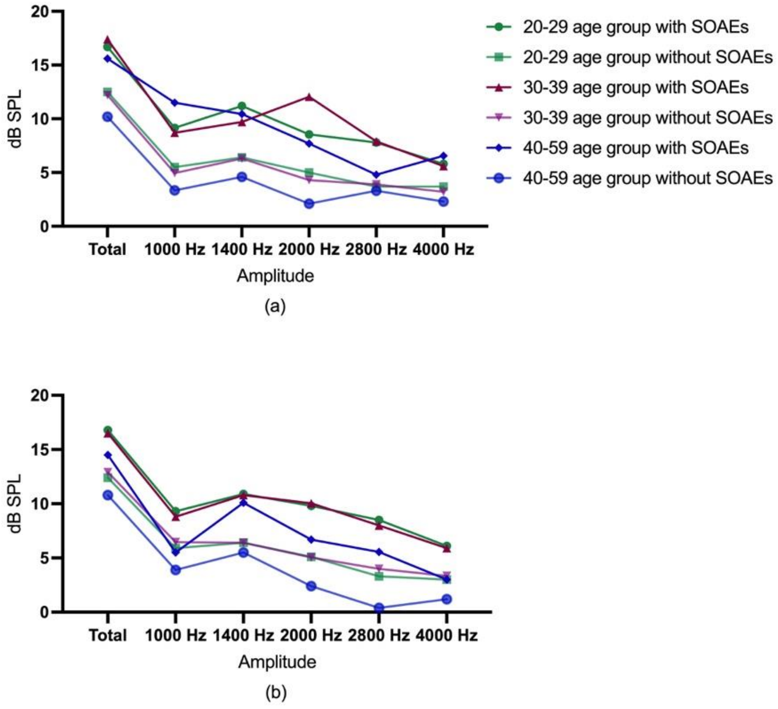

Figure S3.1. a, median TEOAE amplitude of left ear across age group for participants with SOAEs and without SOAEs. b, median TEOAE amplitude of right ear across age group for participants with SOAEs and without SOAEs.

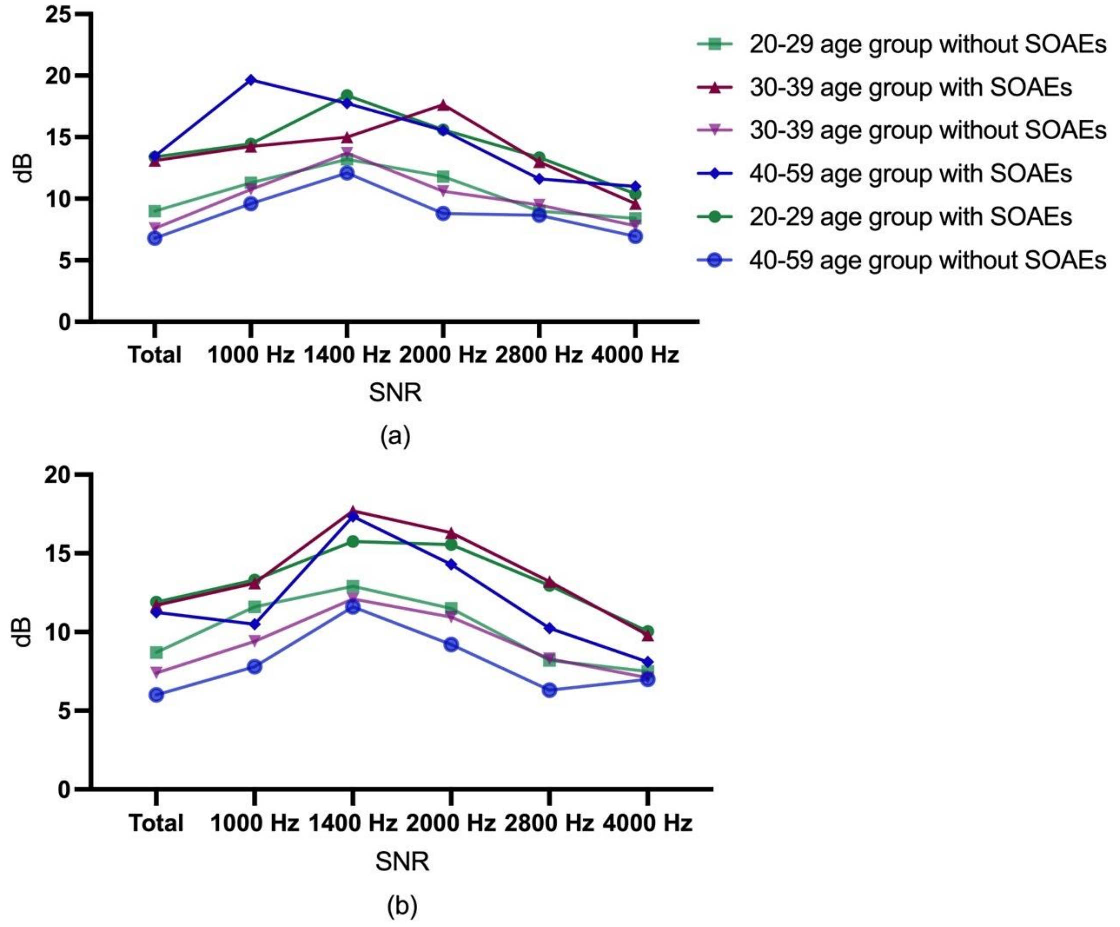

Figure S3.2. a, median TEOAE SNR of left ear across age group for participants with SOAEs and without SOAEs. b, median TEOAE SNR of right ear across age group for participants with SOAEs and without SOAEs.

Supplementary Figure S4:

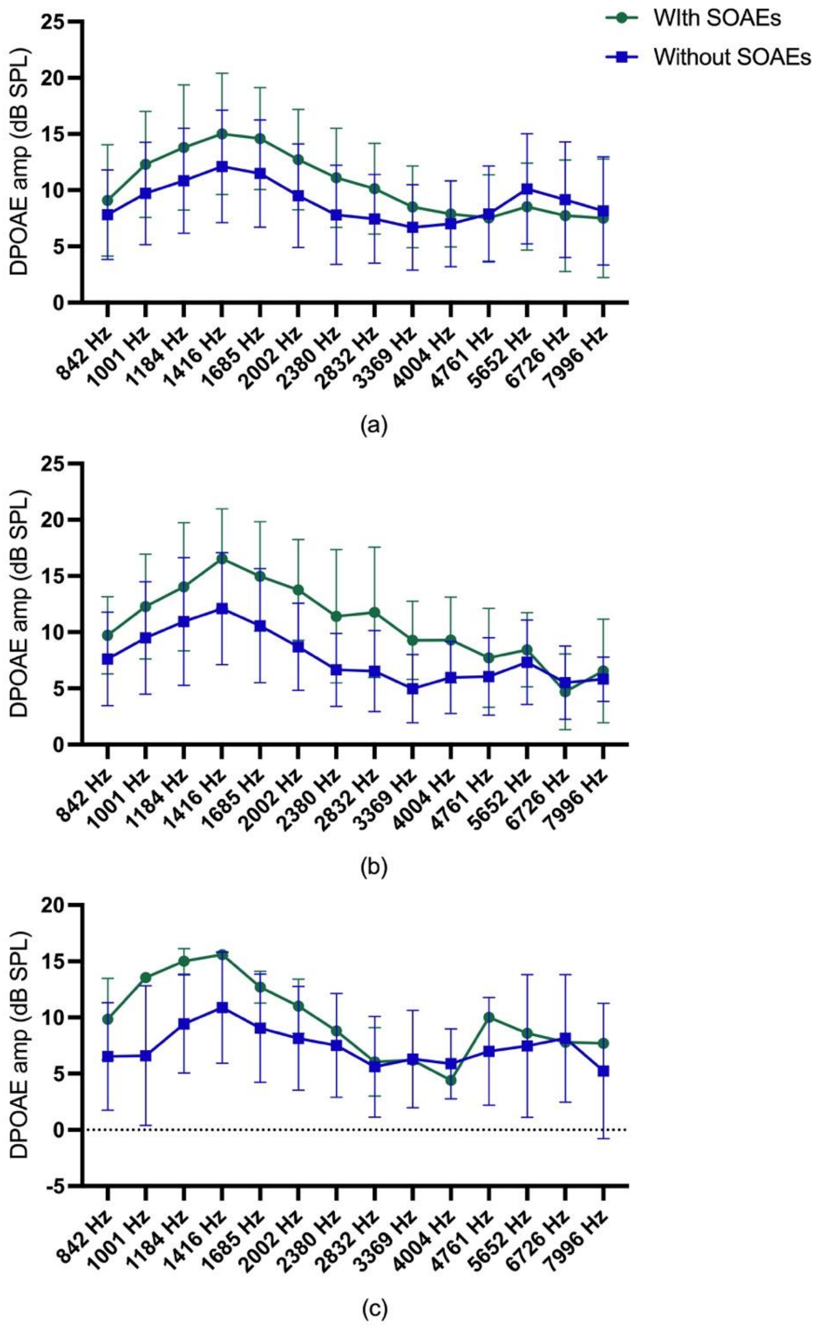

Figure S4. a, mean and standard deviation of DPOAE amplitude of left ear of male participants with and without SOAEs in 20-29 age group; b, mean and standard deviation of DPOAE amplitude of left ear of male participants with and without SOAEs in 30-39 age group; c, mean and standard deviation of DPOAE amplitude of left ear of male participants with and without SOAEs in 40-59 age group.

Supplementary Figure S5:

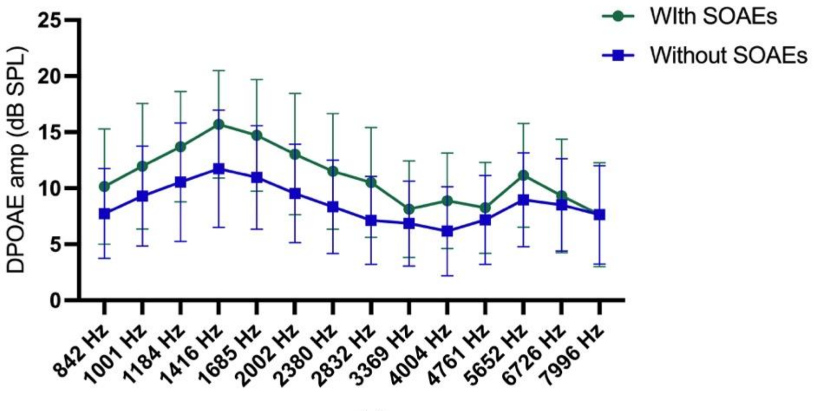

(a)

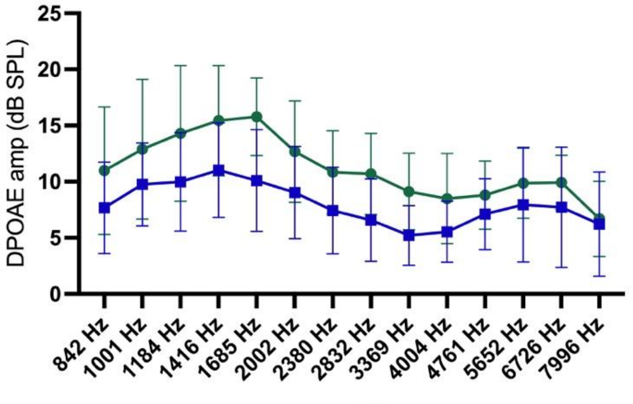

(b)

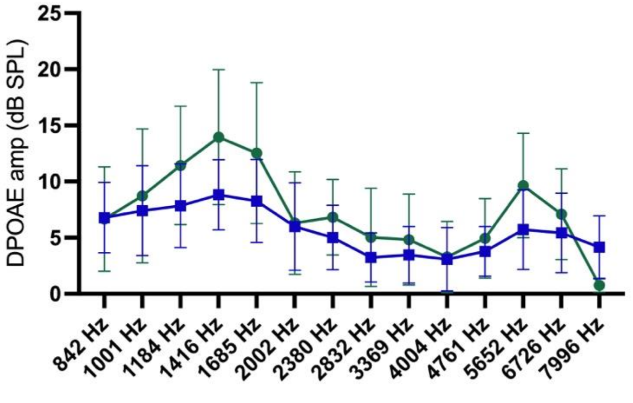

(c)

Figure S5. a, mean and standard deviation of DPOAE amplitude of left ear of female participants with and without SOAEs in 20-29 age group; b, mean and standard deviation of DPOAE amplitude of left ear of female participants with and without SOAEs in 30-39 age group; c, mean and standard deviation of DPOAE amplitude of left ear of female participants with and without SOAEs in 40-59 age group.

Supplementary table S2:

Left TEOAE amplitude

A multiple regression was run to explain total TEOAE amplitude from age, gender and SOAE status. The multiple regression model statistically significant in explaining TEOAE amplitude, $F(3, 430) = 77.71$, $p < .0005$, adj $R^2 = 0.35$. Age, gender and SOAE status added statistically significantly to the explanation, $p < .001$. Regression coefficients and standard errors can be found in table below.

TEOAE	*B*	95 % CI for *B*		*SE B*	β	R^2	ΔR^2
		LL	*UL*				
Model						0.35	0.35***
Constant	13.02***	11.71	14.34	.67			
Age	-.007***	-.01	-.004	.002	-.17		
Gender	1.06**	.45	1.66	.31	.14		
SOAE	4.25***	3.61	4.89	.33	.52		

Note. Model = “Enter” method in SPSS Statistics; *B* = unstandardized regression coefficient; CI = confidence interval; *LL* = lower limit; *UL* = upper limit; *SE B* = standard error of the coefficient; β = standardized coefficient; R^2 = coefficient of determination; ΔR^2 = adjusted ΔR^2.
*p < .05. **p < .01. ***p< .0005
Note. The table that follows share this note.

Left TEOAE SNR

A multiple regression was run to explain total TEOAE SNR from age, gender and SOAE status. The multiple regression model statistically significant in explaining TEOAE SNR, $F(3, 430) = 58.97$, $p < .0005$, adj $R^2 = 0.29$. Age, gender and SOAE status added statistically significantly to the explanation, $p < .001$. Regression coefficients and standard errors can be found in table below.

TEOAE	*B*	95 % CI for *B*		*SE B*	β	R^2	ΔR^2
		LL	*UL*				
Model						0.29	0.29***
Constant	9.65***	8.22	11.09	.73			
Age	-.007***	-.01	-.003	.002	-.16		
Gender	.97**	.31	1.63	.34	.12		
SOAE	4.02***	3.32	4.71	.35	.47		

Supplementary table S3:

Right TEOAE amplitude

A multiple regression was run to explain total TEOAE amplitude from age, gender and SOAE status. The multiple regression model statistically significant in explaining TEOAE amplitude, $F(3, 442)= 59.49$, $p < .0005$, adj $R^2= 0.28$. Age, gender and SOAE status added statistically significantly to the explanation, $p < .001$. Regression coefficients and standard errors can be found in table below.

TEOAE	*B*	95 % CI for *B*		*SE B*	β	R^2	ΔR^2
		LL	*UL*				
Model						0.29	0.28***
Constant	13.03***	11.56	14.50	.75			
Age	-.006***	-.009	-.003	.002	-.14		
Gender	1.06**	.40	1.72	.34	.13		
SOAE	3.95***	3.30	4.61	.34	.48		

Right TEOAE SNR

A multiple regression was run to explain total TEOAE SNR from age, gender and SOAE status. The multiple regression model statistically significant in explaining TEOAE SNR, $F(3, 442)= 42.36$, $p < .0005$, adj $R^2= 0.22$. Age and SOAE status added statistically significantly to the explanation, $p < .001$. Regression coefficients and standard errors can be found in table below.

TEOAE	*B*	95 % CI for *B*		*SE B*	β	R^2	ΔR^2
		LL	*UL*				
Model						0.22	0.22***
Constant	9.03***	7.51	10.56	.78			
Age	-.005**	-.008	-.001	.002	-.11		
Gender	.56	-.12	1.25	.35	.07		
SOAE	3.61***	2.92	4.29	.35	.44		

Table S4. Spearman correlation test results between TEOAEs and age in left and right ears.

**Left**	**Total amplitude**	**Total SNR**	**Right**	**Total amplitude**	**Total SNR**
With SOAE	-0.037	-0.032	With SOAE	-0.068	0.010
	NS	NS		NS	NS
Without SOAE	-0.211	-0.252	Without SOAE	-0.146	-0.148
	***	***		*	*

Note: NS indicates not significant, asterisk * indicates $p < 0.05$, asterisk ** indicates $p < 0.001$, asterisk *** indicates $p < 0.0005$.

Supplementary table S5:

Left DPOAE amplitude (overall average frequencies)

A multiple regression was run to explain DPOAE amplitude at overall average frequencies from age, gender and SOAE status. The multiple regression model statistically significant in explaining DPOAE amplitude, $F(3, 425)= 26.180$, $p < .0005$, adj $R^2= 0.15$. Age and SOAE status added statistically significantly to the explanation, $p < .0005$. Regression coefficients and standard errors can be found in table below.

DPOAE	*B*	95 % CI for *B*		*SE B*	β	R^2	ΔR^2
		LL	*UL*				
Model						0.16	0.15***
Constant	10.91***	9.41	12.41	.76			
Age	-.01***	-.01	-.006	.002	-.24		
Gender	-.27	-.93	.39	.34	-.03		
SOAE	2.21***	1.52	2.91	.35	.29		

Left DPOAE SNR (overall average frequencies)

A multiple regression was run to explain DPOAE SNR at overall average frequencies from age, gender and SOAE status. The multiple regression model statistically significant in explaining DPOAE SNR, $F(3, 425)= 17.647$, $p < .0005$, adj $R^2= 0.10$. Age and SOAE status added statistically significantly to the explanation, $p < .0005$. Regression coefficients and standard errors can be found in table below.

DPOAE	*B*	95 % CI for *B*		*SE B*	β	R^2	ΔR^2
		LL	*UL*				
Model						0.11	0.10***
Constant	19.31***	17.49	21.12	.92			
Age	-.01***	-.01	-.006	.002	-.20		
Gender	-.33	-1.13	.47	.41	-.03		
SOAE	2.18***	1.33	3.02	.43	.24		

Left DPOAE amplitude (average low frequencies)

A multiple regression was run to explain DPOAE amplitude at average low frequencies from age, gender and SOAE status. The multiple regression model statistically significant in explaining DPOAE amplitude, $F(3, 431)= 22.937$, $p < .0005$, adj $R^2= 0.13$. Age and SOAE status added statistically significantly to the explanation, $p < .0005$. Regression coefficients and standard errors can be found in table below.

DPOAE	*B*	95 % CI for *B*		*SE B*	β	R^2	ΔR^2
		LL	*UL*				
Model						0.14	0.13***
Constant	12.46***	10.65	14.26	.92			
Age	-.007***	-.01	-.003	.002	-.16		
Gender	-.23	-1.07	.59	.42	-.02		
SOAE	3.07***	2.19	3.96	.45	.31		

Note. Model = "Enter" method in SPSS Statistics; *B* = unstandardized regression coefficient; CI = confidence interval; *LL* = lower limit; *UL* = upper limit; *SE B* = standard error of the coefficient; β = standardized coefficient; R^2 = coefficient of determination; ΔR^2 = adjusted ΔR^2.
*p < .05. **p < .01. ***p< .0005

Left DPOAE SNR (average low frequencies)

A multiple regression was run to explain DPOAE SNR at average low frequencies from age, gender and SOAE status. The multiple regression model statistically significant in explaining DPOAE SNR, $F(3, 431)= 11.975$, $p < .0005$, adj $R^2= 0.077$. Age and SOAE status added statistically significantly to the explanation, $p < .001$. Regression coefficients and standard errors can be found in table below.

DPOAE	*B*	95 % CI for *B*		*SE B*	β	R^2	ΔR^2
		LL	*UL*				
Model						0.08	0.07***
Constant	16.16***	13.75	18.57	1.23			
Age	-.008**	-.01	-.003	.003	-.14		
Gender	-.28	-1.39	.83	.57	-.02		
SOAE	2.78***	1.59	3.97	.60	.22		

Note. Model = "Enter" method in SPSS Statistics; *B* = unstandardized regression coefficient; CI = confidence interval; *LL* = lower limit; *UL* = upper limit; *SE B* = standard error of the coefficient; β = standardized coefficient; R^2 = coefficient of determination; ΔR^2 = adjusted ΔR^2.
*p < .05. **p < .01. ***p< .0005

Left DPOAE amplitude (average mid frequencies)

A multiple regression was run to explain DPOAE amplitude at average mid frequencies from age, gender and SOAE status. The multiple regression model statistically significant in explaining DPOAE amplitude, $F(3, 413)= 28.92$, $p < .0005$, adj $R^2= 0.174$. Age and SOAE status added statistically significantly to the explanation, $p < .0005$. Regression coefficients and standard errors can be found in table below.

DPOAE	*B*	95 % CI for *B*		*SE B*	β	R^2	ΔR^2
		LL	*UL*				
Model						0.17	0.17***
Constant	10.47***	8.85	12.08	.82			
Age	-.01***	-.01	-.007	.002	-.24		
Gender	-.18	-.88	.52	.36	-.02		
SOAE	2.60***	1.86	3.34	.38	.32		

Note. Model = “Enter” method in SPSS Statistics; *B* = unstandardized regression coefficient; CI = confidence interval; *LL* = lower limit; *UL* = upper limit; *SE B* = standard error of the coefficient; β = standardized coefficient; R^2 = coefficient of determination; ΔR^2 = adjusted ΔR^2.
*p < .05. **p < .01. ***p< .0005

Left DPOAE SNR (average mid frequencies)

A multiple regression was run to explain DPOAE SNR at average mid frequencies from age, gender and SOAE status. The multiple regression model statistically significant in explaining DPOAE SNR, $F(3, 413)= 23.06$, $p < .0005$, adj $R^2= 0.143$. Age and SOAE status added statistically significantly to the explanation, $p < .0005$. Regression coefficients and standard errors can be found in table below.

DPOAE	*B*	95 % CI for *B*		*SE B*	β	R^2	ΔR^2
		LL	*UL*				
Model						0.14	0.14***
Constant	20.69***	18.89	22.50	.92			
Age	-.01***	-.01	-.005	.002	-.20		
Gender	-.35	-1.14	.44	.40	-.04		
SOAE	2.72***	1.89	3.54	.42	.29		

Note. Model = “Enter” method in SPSS Statistics; *B* = unstandardized regression coefficient; CI = confidence interval; *LL* = lower limit; *UL* = upper limit; *SE B* = standard error of the coefficient; β = standardized coefficient; R^2 = coefficient of determination; ΔR^2 = adjusted ΔR^2.
*p < .05. **p < .01. ***p< .0005

Left DPOAE amplitude (average high frequencies)

A multiple regression was run to explain DPOAE amplitude at average high frequencies from age, gender and SOAE status. The multiple regression model statistically significant in explaining DPOAE amplitude, $F(3, 367)= 4.524$, $p = .004$, adj $R^2= 0.04$. Age added statistically significantly to the explanation, $p < .0005$. Regression coefficients and standard errors can be found in table below.

DPOAE	*B*	95 % CI for *B*		*SE B*	β	R^2	ΔR^2
		LL	*UL*				
Model						0.04	0.03**
Constant	10.28***	8.22	12.33	1.05			
Age	-.01***	-.01	-.004	.003	-.18		
Gender	.11	-.74	.96	.43	.01		
SOAE	.38	-.50	1.27	.45	.04		

Note. Model = "Enter" method in SPSS Statistics; *B* = unstandardized regression coefficient; CI = confidence interval; *LL* = lower limit; *UL* = upper limit; *SE B* = standard error of the coefficient; β = standardized coefficient; R^2 = coefficient of determination; ΔR^2 = adjusted ΔR^2.
*p < .05. **p < .01. ***p< .0005

Left DPOAE SNR (average high frequencies)

A multiple regression was run to explain DPOAE SNR at average high frequencies from age, gender and SOAE status. The multiple regression model statistically significant in explaining DPOAE SNR, $F(3, 367)= 3.670$, $p = .012$, adj $R^2= 0.03$. Age added statistically significantly to the explanation, $p < .0005$. Regression coefficients and standard errors can be found in table below.

DPOAE	*B*	95 % CI for *B*		*SE B*	β	R^2	ΔR^2
		LL	*UL*				
Model						0.03	0.02*
Constant	21.98***	19.83	24.14	1.10			
Age	-.009***	-.01	-.003	.003	-.15		
Gender	.24	-.65	1.13	.45	.03		
SOAE	.57	-.36	1.49	.47	.06		

Note. Model = "Enter" method in SPSS Statistics; *B* = unstandardized regression coefficient; CI = confidence interval; *LL* = lower limit; *UL* = upper limit; *SE B* = standard error of the coefficient; β = standardized coefficient; R^2 = coefficient of determination; ΔR^2 = adjusted ΔR^2.
*p < .05. **p < .01. ***p< .0005

Supplementary table S6:

Right DPOAE amplitude (overall average frequencies)

A multiple regression was run to explain DPOAE amplitude at overall average frequencies from age, gender and SOAE status. The multiple regression model statistically significant in explaining DPOAE amplitude, $F(3, 431)= 29.47$, $p < .0005$, adj $R^2= 0.16$. Age and SOAE status added statistically significantly to the explanation, $p < .001$. Regression coefficients and standard errors can be found in table below.

DPOAE	*B*	95 % CI for *B*		*SE B*	β	R^2	ΔR^2
		LL	*UL*				
Model						0.17	0.16***
Constant	11.10***	9.59	12.62	.77			
Age	-.01**	-.01	-.008	.002	-.27		
Gender	-.05	-.61	.71	.34	-.006		
SOAE	2.16***	1.50	2.82	.34	.29		

Note. Model = "Enter" method in SPSS Statistics; *B* = unstandardized regression coefficient; CI = confidence interval; *LL* = lower limit; *UL* = upper limit; *SE B* = standard error of the coefficient; β = standardized coefficient; R^2 = coefficient of determination; ΔR^2 = adjusted ΔR^2.
*p < .05. **p < .01. ***p< .0005

Right DPOAE SNR (overall average frequencies)

A multiple regression was run to explain DPOAE SNR at overall average frequencies from age, gender and SOAE status. The multiple regression model statistically significant in explaining DPOAE SNR, $F(3, 431)= 17.34$, $p < .0005$, adj $R^2= 0.10$. Age and SOAE status added statistically significantly to the explanation, $p < .001$. Regression coefficients and standard errors can be found in table below.

DPOAE	*B*	95 % CI for *B*		*SE B*	β	R^2	ΔR^2
		LL	*UL*				
Model						0.11	0.10***
Constant	18.02***	16.17	19.87	.94			
Age	-.01**	-.01	-.006	.002	-.20		
Gender	-.10	-.91	.70	.41	-.012		
SOAE	2.14***	1.34	2.95	.41	.24		

Note. Model = "Enter" method in SPSS Statistics; *B* = unstandardized regression coefficient; CI = confidence interval; *LL* = lower limit; *UL* = upper limit; *SE B* = standard error of the coefficient; β = standardized coefficient; R^2 = coefficient of determination; ΔR^2 = adjusted ΔR^2.
*p < .05. **p < .01. ***p< .0005

Right DPOAE amplitude (average low frequencies)

A multiple regression was run to explain DPOAE amplitude at average low frequencies from age, gender and SOAE status. The multiple regression model statistically significant in explaining DPOAE amplitude, $F(3, 446)= 20.35$, $p < .0005$, adj $R^2= 0.11$. Age and SOAE status added statistically significantly to the explanation, $p < .001$. Regression coefficients and standard errors can be found in table below.

DPOAE	*B*	95 % CI for *B*		*SE B*	β	R^2	ΔR^2
		LL	*UL*				
Model						0.12	0.11***
Constant	12.93***	11.09	14.76	.94			
Age	-.007**	-.01	-.003	.002	-.15		
Gender	-.63	-1.46	.21	.43	-.06		
SOAE	2.77***	1.94	3.61	.43	.29		

Note. Model = "Enter" method in SPSS Statistics; *B* = unstandardized regression coefficient; CI = confidence interval; *LL* = lower limit; *UL* = upper limit; *SE B* = standard error of the coefficient; β = standardized coefficient; R^2 = coefficient of determination; ΔR^2 = adjusted ΔR^2.
*p < .05. **p < .01. ***p< .0005

Right DPOAE SNR (average low frequencies)

A multiple regression was run to explain DPOAE SNR at average low frequencies from age, gender and SOAE status. The multiple regression model statistically significant in explaining DPOAE SNR, $F(3, 446)= 9.23$, $p < .0005$, adj $R^2= 0.05$. SOAE status added statistically significantly to the explanation, $p < .0005$. Regression coefficients and standard errors can be found in table below.

DPOAE	*B*	95 % CI for *B*		*SE B*	β	R^2	ΔR^2
		LL	*UL*				
Model						0.06	0.05***
Constant	14.06***	11.57	16.55	1.27			
Age	-.005	-.01	0.00	.003	-.09		
Gender	-.96	-2.09	.17	.58	-.07		
SOAE	2.54***	1.41	3.67	.58	.21		

Note. Model = "Enter" method in SPSS Statistics; *B* = unstandardized regression coefficient; CI = confidence interval; *LL* = lower limit; *UL* = upper limit; *SE B* = standard error of the coefficient; β = standardized coefficient; R^2 = coefficient of determination; ΔR^2 = adjusted ΔR^2.
*p < .05. **p < .01. ***p< .0005

Right DPOAE amplitude (average mid frequencies)

A multiple regression was run to explain DPOAE amplitude at average mid frequencies from age, gender and SOAE status. The multiple regression model statistically significant in explaining DPOAE amplitude, *F*(3, 417)= 27.59, $p < .0005$, adj R^2= 0.16. Age and SOAE status added statistically significantly to the explanation, $p < .0005$. Regression coefficients and standard errors can be found in table below.

DPOAE	*B*	95 % CI for *B*		*SE B*	β	R^2	ΔR^2
		LL	*UL*				
Model						0.17	0.16***
Constant	10.51***	8.81	12.21	.87			
Age	-.01***	-.01	-.006	.002	-.21		
Gender	-.06	-.79	.66	.37	-.008		
SOAE	2.69***	1.97	3.42	.37	.33		

Note. Model = “Enter” method in SPSS Statistics; *B* = unstandardized regression coefficient; CI = confidence interval; *LL* = lower limit; *UL* = upper limit; *SE B* = standard error of the coefficient; β = standardized coefficient; R^2 = coefficient of determination; ΔR^2 = adjusted ΔR^2.
*p < .05. **p < .01. ***p< .0005

Right DPOAE SNR (average mid frequencies)

A multiple regression was run to explain DPOAE SNR at average mid frequencies from age, gender and SOAE status. The multiple regression model statistically significant in explaining DPOAE SNR, *F*(3, 417)= 19.95, $p < .0005$, adj R^2= 0.12. Age and SOAE status added statistically significantly to the explanation, $p < .0005$. Regression coefficients and standard errors can be found in table below.

DPOAE	*B*	95 % CI for *B*		*SE B*	β	R^2	ΔR^2
		LL	*UL*				
Model						0.13	0.12***
Constant	19.40***	17.44	21.36	1.00			
Age	-.008**	-.01	-.003	.002	-.15		
Gender	-.23	-1.07	.60	.43	-.02		
SOAE	2.83***	1.99	3.66	.43	.31		

Note. Model = “Enter” method in SPSS Statistics; *B* = unstandardized regression coefficient; CI = confidence interval; *LL* = lower limit; *UL* = upper limit; *SE B* = standard error of the coefficient; β = standardized coefficient; R^2 = coefficient of determination; ΔR^2 = adjusted ΔR^2.
*p < .05. **p < .01. ***p< .0005

Right DPOAE amplitude (average high frequencies)

A multiple regression was run to explain DPOAE amplitude at average high frequencies from age, gender and SOAE status. The multiple regression model statistically significant in explaining DPOAE amplitude, $F(3, 379)= 7.56$, $p < .0005$, adj $R^2= 0.05$. Age and SOAE status added statistically significantly to the explanation, $p < .05$. Regression coefficients and standard errors can be found in table below.

DPOAE	*B*	95 % CI for *B*		*SE B*	β	R^2	ΔR^2
		LL	*UL*				
Model						0.06	0.05***
Constant	10.57***	8.57	12.56	1.01			
Age	-.01***	-.01	-.006	.003	-.20		
Gender	-.13	-.98	.71	.43	-.01		
SOAE	.90*	.06	1.73	.43	.11		

Note. Model = "Enter" method in SPSS Statistics; *B* = unstandardized regression coefficient; CI = confidence interval; *LL* = lower limit; *UL* = upper limit; *SE B* = standard error of the coefficient; β = standardized coefficient; R^2 = coefficient of determination; ΔR^2 = adjusted ΔR^2.
*p < .05. **p < .01. ***p< .0005

Right DPOAE SNR (average high frequencies)

A multiple regression was run to explain DPOAE SNR at average high frequencies from age, gender and SOAE status. The multiple regression model statistically significant in explaining DPOAE SNR, $F(3, 379)= 5.91$, $p = .001$, adj $R^2= 0.04$. Age and SOAE status added statistically significantly to the explanation, $p < .05$. Regression coefficients and standard errors can be found in table below.

DPOAE	*B*	95 % CI for *B*		*SE B*	β	R^2	ΔR^2
		LL	*UL*				
Model						0.05	0.04**
Constant	22.07***	19.91	24.22	1.10			
Age	-.01**	-.01	-.004	.003	-.17		
Gender	-.001	-.91	.92	.47	0.00		
SOAE	1.03*	.13	1.94	.46	.11		

Note. Model = "Enter" method in SPSS Statistics; *B* = unstandardized regression coefficient; CI = confidence interval; *LL* = lower limit; *UL* = upper limit; *SE B* = standard error of the coefficient; β = standardized coefficient; R^2 = coefficient of determination; ΔR^2 = adjusted ΔR^2.
*p < .05. **p < .01. ***p< .0005

Supplementary table S7

Left average extended high frequency hearing threshold

A multiple regression was run to explain EHF hearing threshold from age, gender and SOAE status. The multiple regression model statistically significant in explaining EHF hearing threshold, $F(3, 455)= 153.67$, $p < .0005$, adj $R^2= 0.50$. Age added statistically significantly to the explanation, $p < .0005$. Regression coefficients and standard errors can be found in table below.

EHF	*B*	95 % CI for *B*		*SE B*	β	R^2	ΔR^2
		LL	*UL*				
Model						0.50	0.50***
Constant	-18.68***	-22.51	-14.86	1.95			
Age	.09***	.08	.10	.004	.71		
Gender	-.38	-2.13	1.36	.89	-.01		
SOAE	-.85	-2.73	1.02	.96	-.03		

Note. Model = "Enter" method in SPSS Statistics; *B* = unstandardized regression coefficient; CI = confidence interval; *LL* = lower limit; *UL* = upper limit; *SE B* = standard error of the coefficient; β = standardized coefficient; R^2 = coefficient of determination; ΔR^2 = adjusted ΔR^2.
*p < .05. **p < .01. ***p< .0005

Right average extended high frequency hearing threshold

A multiple regression was run to explain EHF hearing threshold from age, gender and SOAE status. The multiple regression model statistically significant in explaining EHF hearing threshold, $F(3, 452)= 119.21$, $p < .0005$, adj $R^2= 0.44$. Age added statistically significantly to the explanation, $p < .0005$. Regression coefficients and standard errors can be found in table below.

EHF	*B*	95 % CI for *B*		*SE B*	β	R^2	ΔR^2
		LL	*UL*				
Model						0.44	0.44***
Constant	-16.95***	-20.98	-12.92	2.05			
Age	.09***	.08	.10	.005	.66		
Gender	.10	-1.67	1.88	.91	.004		
SOAE	-.21	-1.99	1.57	.91	-.008		

Note. Model = "Enter" method in SPSS Statistics; *B* = unstandardized regression coefficient; CI = confidence interval; *LL* = lower limit; *UL* = upper limit; *SE B* = standard error of the coefficient; β = standardized coefficient; R^2 = coefficient of determination; ΔR^2 = adjusted ΔR^2.
*p < .05. **p < .01. ***p< .0005
